# Supplementary material for: Erythritol Ameliorates Small Intestinal Inflammation Induced by High-Fat Diets and Improves Glucose Tolerance
Source: Int J Mol Sci. 2021 May 24;22(11):5558. doi: 10.3390/ijms22115558 (PMC8197374; doi:10.3390/ijms22115558)
Supplement: Supplementary file 1 [file ijms-22-05558-s001.zip › Ery supplementary figures.pdf]

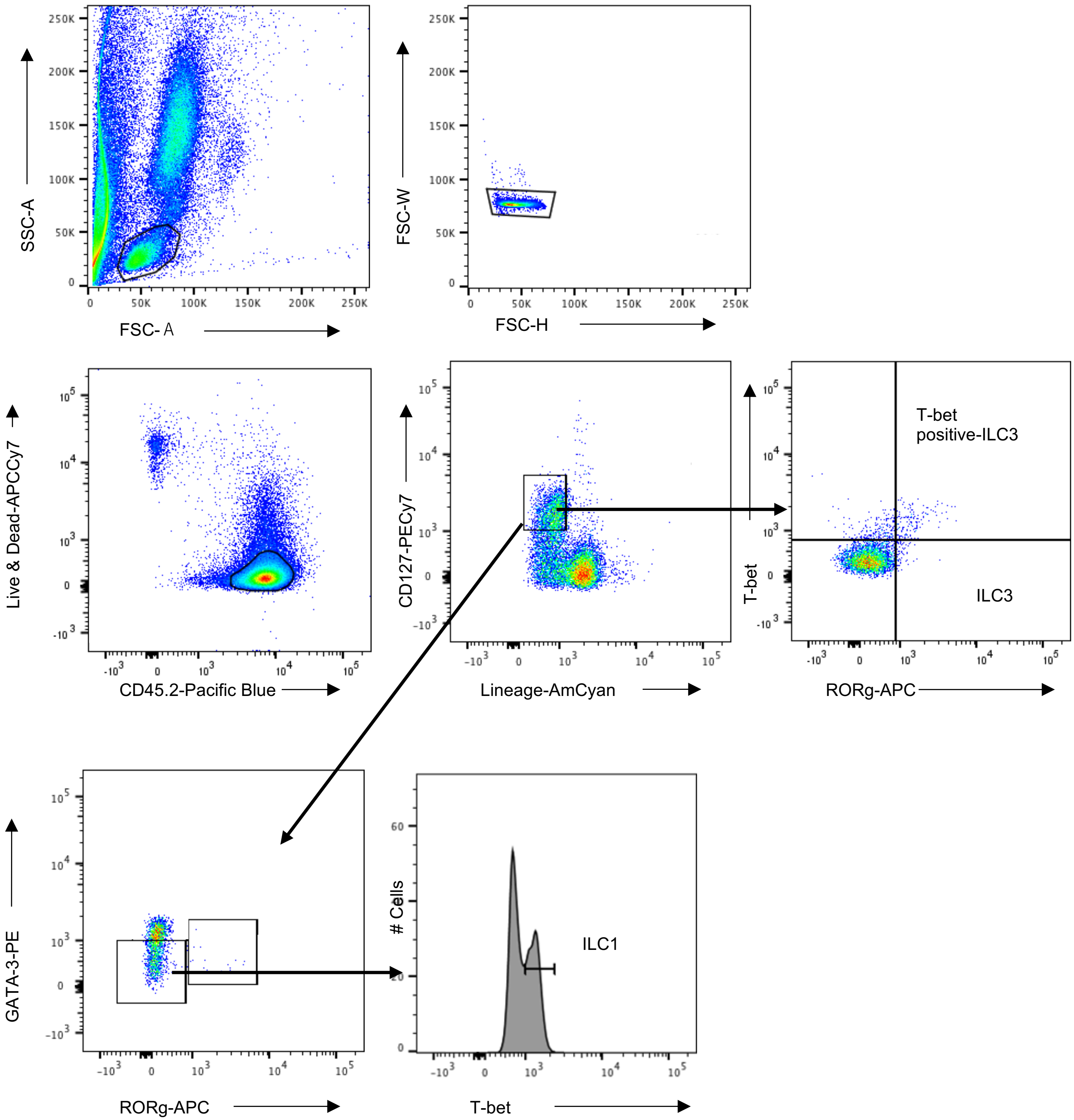

Supplementary Figure 1. Strategy for innate lymphoid cells (ILCs)

Representative flow cytometry plots of liver CD45+ Live & Dead- Lin- CD127+ RORg- GATA-3- T-bet+ ILC1s, CD45+ Live & Dead- Lin- CD127+ RORg+ T-bet- ILC3s and CD45+ Live & Dead- Lin- CD127+ RORg+ T-bet+ T-bet positive ILC3s in each group at 20-weeks of age.

Supplementary Fig. 1

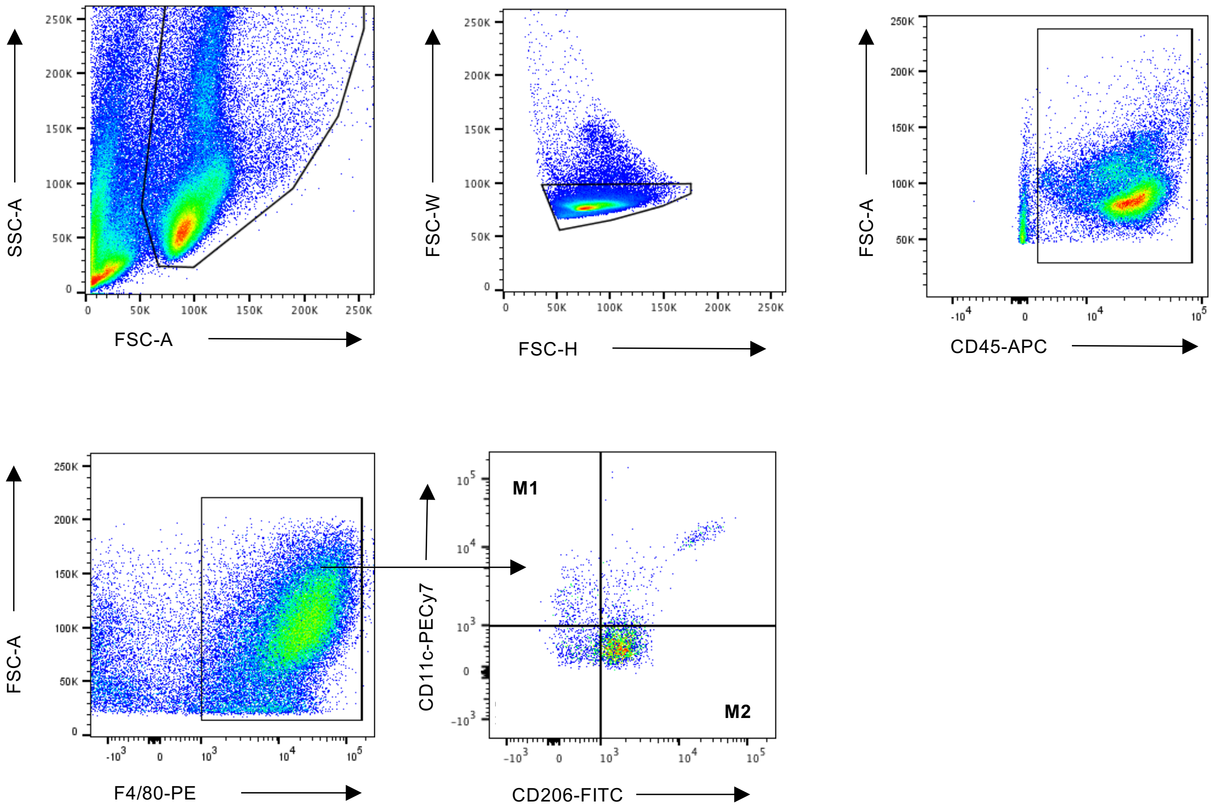

Supplementary Figure 2. Strategy for macrophages

Representative flow cytometry plots of WAT CD45+ F4/80+ CD206- CD11c+ M1 macrophages and CD45+ F4/80+ CD206+ CD11c- M2 macrophages in each group at 20 weeks of age.

Supplementary Fig. 2
