# Supplementary material for: Erythritol Ameliorates Small Intestinal Inflammation Induced by High-Fat Diets and Improves Glucose Tolerance
Source: Int J Mol Sci. 2021 May 24;22(11):5558. doi: 10.3390/ijms22115558 (PMC8197374; doi:10.3390/ijms22115558)
Supplement: Supplementary file 1 [file ijms-22-05558-s001.zip › Supplementary Table 1.pdf]

Antibody	Clone	Vendor	Dilution
biotin-CD3e	145-2C11	Biolegend	1:200
biotin-CD45R/B220	RA3–6B2	Biolegend	1:200
biotin-Gr-1	RB6-8C5	Biolegend	1:200
biotin-CD11c	N418	Biolegend	1:200
biotin-CD11b	M1/70	Biolegend	1:200
biotin-Ter119	TER-119	Biolegend	1:200
biotin-FceRIa	MAR-1	Biolegend	1:200
FITC-streptavidin	-	Biolegend	1:500
PE-Cy7-CD127	A7R34	Biolegend	1:100
Pacific Blue-CD45	30-F11	Biolegend	1:100
PE-GATA-3	TWAJ	Invitrogen	1:50
APC-RORγ	AFKJS-9	Invitrogen	1:50
eFluor 780- Fixable Viability Dye	-	Invitrogen	1:400
APC-CD45.2	104	Invitrogen	1:50
PE-F4/80	BM8	Invitrogen	1:50
APC-Cy7-CD11b	M1/70	Biolegend	1:50
FITC-CD206	MR5D3	Invitrogen	1:50
PE-Cy7-CD11c	N418	Invitrogen	1:50
